# Trends in physical health complaints among adolescents from 2014 – 2019: Considering screen time, social media use, and physical activity

**DOI:** 10.1016/j.ssmph.2023.101394

**Published:** 2023-04-11

**Authors:** Sondre Aasen Nilsen, Kjell Morten Stormark, Ove Heradstveit, Kyrre Breivik

**Affiliations:** a Regional Centre for Child and Youth Mental Health and Child Welfare, NORCE Norwegian Research Centre, Postbox 22, Nygårdstangen, 5838, Bergen, Norway; b Department of Health Promotion, Norwegian Institute of Public Health, Bergen, Norway; c Centre for Alcohol and Drug Research, Stavanger University Hospital, Stavanger, Norway

**Keywords:** Adolescence, Physical health complaints, Screen time, Social media, Trends

## Abstract

The rising rates of physical and mental health complaints among adolescents observed in many countries have coincided with an increased time spent on screen-based devices, including social media use. We sought to document recent trends in physical health complaints (PHC) and whether co-occurring trends in screen time, social media use, and physical activity may account for these trends.

To achieve these aims, we used data from the nationwide Ungdata surveys conducted annually at the municipality level in Norway, comprising 419,934 adolescents aged 13–18 from six survey years (2014–2019). Six items assessed PHC, including neck and shoulder pain, headache, and abdominal pain, during the past month. To account for the nesting structure of Ungdata, and to exploit the variation within and between municipalities, we used multilevel analyses with adolescents nested in municipality-years (n = 669), nested in municipalities (n = 345). We found a small to moderate linear increase in number of PHC among boys and girls from 2014 to 2019. Screen time and social media use moderately attenuated the trend for girls, and to a lesser extent for boys. Screen time and social media use were further positively associated with PHC across the between and within-municipality levels, and social media use was more strongly associated with PHC for girls than boys across all levels of analysis. A similar pattern emerged when considering each symptom individually. The results suggest that the prevalence of PHC rose in tandem with a group-level shift towards higher screen time and social media use. Moreover, the results indicate that higher screen time and social media use may have led to changes in the youth culture with potential consequences for adolescents’ well-being.

## Highlights

• Using nationwide data of ∼ 400,000 adolescents, we examined trends in physical health complaints (PHC) from 2014 to 2019.
• Experiencing frequent PHC increased from 2014 to 2019, and more among girls than boys.
• Screen time and social media use attenuated a moderate part of the trend in PHC for girls, and to a lesser extent for boys.
• PHC rose in tandem with a group level shift toward higher screen time and social media use.
• Trends in PHC in Norway may be partly fueled by a population-level shift toward higher screen time and social media use.

## 1 Introduction

Rising rates of physical and mental health complaints among adolescents have been observed in the northern part of Europe and the US (Högberg et al., 2022; Roy et al., 2022; Twenge et al., 2018). When detected, identifying the determinants of these trends is important to inform public health interventions. However, the causes of these trends are still poorly understood. To provide insight into this question, we examine trends in physical health complaints (PHC) as an indicator of adolescents well-being using data from more than 400,000 adolescents across 345 municipalities and 669 municipality-years in Norway. We further examine if changes in screen time, social media use, and physical activity may account for trends in PHC, and whether these putative mechanisms are associated with PHC across the between and within-municipality level of analysis.

PHC such as headache and neck, shoulder, and back pain, are common in adolescence (Myrtveit Sæther et al., 2018; Swain et al., 2014). Females report more PHC than males; a pattern found to be consistent across countries and continents (Gobina et al., 2019). Although PHC may stem from a physical injury or an underlying disease, many adolescents experience them without any known medical condition (Eminson, 2007). PHC are associated with poorer academic achievement (Låftman & Magnusson, 2017) and are prognostic of hospital contacts during adolescence (Bernstorff et al., 2022) and psychiatric diagnosis in adulthood (Bohman et al., 2012). Musculoskeletal pain and headache are among the leading causes of years lived with disabilities in adolescence and young adulthood (Vos et al., 2016), highlighting the importance of monitoring trends in such symptoms from a public health perspective.

Using data covering 1991 to 2018, research from Denmark (Holstein et al., 2018, 2021, 2022), Finland (Ståhl et al., 2014), and a cross-national study of European countries (Roy et al., 2022), suggest that recurrent abdominal pain, back pain, neck pain, and headache have all become more prevalent in recent years in the adolescent population. PHC are also frequently combined with measures of anxiety and depression, often labeled psychosomatic- or simply (subjective) health complaints in the literature. Studies using such measures have yielded similar findings (Ottova-Jordan et al., 2015; Potrebny et al., 2019). The trends appear strongest among adolescent girls (Potrebny et al., 2017), but little is known about trends among adolescents above 16 years of age.

The search for the determinants of these trends has been intensified. Studies have examined the influence of school stress (Cosma et al., 2020; Högberg et al., 2020), bullying, alcohol and drug use, and parent- and peer relations (De Looze et al., 2020). School stress has been highlighted as the most promising one (Cosma et al., 2020; Högberg et al., 2020). However, neither school stress alone, nor combined with other factors, have been found to explain more than a small part of the time trends (Cosma et al., 2020). Previous work into the determinants has mostly focused on explaining trends in measures of psychosomatic or emotional symptoms. Studies focusing on PHC are generally lacking.

The last decade has witnessed a sharp rise in the use of screen-based devices and in particular the widespread use of social media (Ghekiere et al., 2019; Twenge et al., 2019). Consequently, screen time (*time spent on screen-based devices)* and social media use have been highlighted as alternative explanations for the rising trends in adolescents physical and mental health complaints (Cosma et al., 2020; Dey et al., 2015; Tang et al., 2021). Moderately strong evidence for associations between screen time and adolescent obesity, depression, and quality of life exists, according to a review of reviews (Stiglic & Viner, 2019). Screen time has also been associated with PHC (Keane et al., 2017; Myrtveit et al., 2014), potentially through sustained muscle tension due to the ergonomic aspects of such activities (Torsheim et al., 2010). In addition, social media has been suggested to induce stress due to self-presentation, approval anxiety, fear of missing out, and information overload (Wolfers & Utz, 2022). Emerging evidence suggest that social media use is associated with PHC but few studies have examined such associations (Lee et al., 2022).

Longitudinal studies tend to find weaker associations between digital technology on indicators of adolescent well-being than cross-sectional studies. Based on this observation, a recent review concluded that the effect of screen time and social media use on current trends in mental health complaints is likely negligible, based on an overall linear estimate of *r* = 0.10 (Tang et al., 2021). However, even small linear relationships between digital technology and adolescent mental health may exist alongside important risk associations between groups (e.g., low vs high users) and thus have practical importance (Twenge & Hamilton, 2022).

The conclusion reached by the above mentioned review might also be an example of committing the atomistic fallacy (c.f., Diez, 2002; Diez-Roux, 1998). That is, using individual-level data to explain group-level trends. Indeed, the determinants of individual cases may not be the same or of similar magnitude as the determinants of incidence rates at the population (Rose, 2001). As such, research of individuals may fail to explain differences between or within regions over time. To examine drivers of population-level trends, data collected at higher-order units, such as municipalities, states, or countries, are needed.

We are only aware of one study examining whether screen time relates to population-level trends in PHC. Using data from the Health Behaviour in School-aged Children (HBSC) study, about 4% of the increased prevalence of back pain from 2002 to 2014 in Europe could be accounted for by rising trends in screen time (Roman-Juan et al., 2022). However, this study only captured the start of the massive expansion in digital media use as smartphones became common and focused only on back pain. Thus, there is a need for more recent studies also examining other symptoms of PHC.

Screen time and social media use may also be uniquely associated with adolescent PHC at the group level. For one, the expansion of the smartphone may have shifted the norms of how adolescents socialize towards less in-person socialization (Twenge et al., 2021). As social interaction is a group-level process, this may influence adolescents independent of their individual level of use. That is, in a context where the average digital media communication is high, the opportunity for in-person socialization is lower for everyone. It has been argued that digital communication permits less emotional closeness than in-person interactions, and existing work has linked these group-level processes to rising trends in loneliness (Twenge et al., 2021). As loneliness often co-occurs with PHC (Lyyra et al., 2018), this shift in the youth culture may also be linked to trends in PHC.

A group-level shift toward higher digital media use could also have led to a youth culture more pressured to succeed in multiple areas of life. Social media tend to portray peoples’ successes more than failures (de Lenne et al., 2020), and the most popular social media accounts tend to be of highly successful professionals. In addition to the effect that upward social comparison may have on the individual level (Nesi & Prinstein, 2015; Schmuck et al., 2019), a group-level shift toward higher social media use may have created a youth culture that is more stressed by the pressures of success. As stress is strongly associated with PHC (Alfven et al., 2008; Wiklund et al., 2012), this may serve as another pathway through which higher levels of digital media use may be linked to rising population-level trends in PHC.

Changing trends in physical activity may also be linked to trends in PHC. The proportion of adolescents not meeting recommended guidelines of physical activity is high (cf., Guthold et al., 2020), and a recent study of Norwegian university students found that physical activity levels might be on a downward trend from 2014 to 2018 (Grasdalsmoen et al., 2019). Less physical activity is associated with higher levels of PHC (Swain et al., 2014). We are, however, unaware of previous work examining whether trends in physical activity could be related to changing trends in PHC, and how physical activity is associated with PHC across the individual- and group levels of analysis.

Although a growing body of research has examined recent trends in various indicators of adolescent well-being, the determinants of these trends are still poorly understood. Existing work has seldom leveraged the potential of their data in understanding how changes in population-level risk factors may be longitudinally related to population trends in adolescents’ well-being (although such methods have recently been used within alcohol research (cf. Halkjelsvik et al., 2021; Vashishtha et al., 2022)). This study examines whether a population-level shift in screen time, social media use, and physical activity is linked with current trends in adolescent PHC. We also examine the partial and interactive effects of screen time, social media use, and physical activity on PHC. To achieve these aims, we take advantage of the variation between and within municipalities over time, and assess whether our putative mechanisms, from the between-individual to the within-municipality level, are associated with PHC among adolescents, and whether the associations vary by gender.

## 2 Methods

### 2.1 Design and procedure

Data stem from the Ungdata surveys, a national data collection scheme at the national and municipal levels in Norway. Ungdata is the most comprehensive source on adolescent health and well-being in Norway (see http://www.ungdata.no/English). Ungdata was started in 2010 and has since 2014 been implemented for all junior high (13–15 year olds) - and high school students (16–19 year olds). Since 2010, most Norwegian municipalities have participated. Data collection was conducted each spring. The average response rate at the individual level across municipalities ranged from 78% to 85% (von Soest et al., 2022). Participants completed an electronic questionnaire in class covering several topics, including physical health complaints and leisure activities.

This study used data from six rounds from 2014 to 2019, with responses from 487,124 adolescents (50% female) nested in 822 municipality years in 416 municipalities. Due to protection of the participants privacy, age and/or gender was not provided for participants in municipalities with fewer than 350 participanting students, amounting to a total of 71 of the 416 municipalities.

In our first set of analyses focusing on the overall time trend by age and gender, the analytical sample consisted of 419,934 adolescents nested in 671 municipality years nested in 345 municipalities, after excluding municipalities and respondents with missing values on age or gender, and respondents with missing values on physical health complaints. The number of participants ranged from 40,414 (in 2014) to 103,973 (in 2019). 91 municipalities had participated one time, 186 municipalities had participated two times, 64 municipalities had participated three times, and four municipalities had participated four times. In our next set of analyses examining the influence of co-occurring trends in screen time, social media use, and physical activity, the number of adolescents was reduced to 402,826 due to individual missing data on either screen time, social media use, or physical activity.

### 2.2 Ethical considerations

This paper reports secondary analysis of existing data. The study followed the Declaration of Helsinki guidelines. All participants and their parents were informed that participation was voluntary. Parents could reserve their children from participation. Data collection from students in grades 8 to 10 was anonymous and did not need approval by data protection agencies. The Norwegian data protection authority approved data collection from students in grades 11 to 13. For more information, see the project web page (https://www.ungdata.no/english/).

### 2.3 Measures

#### 2.3.1 Gender and age

Gender was measured by adolescent self-report. Due to anonymity concerns, age was not explicitly assessed. In Norway, attendance in a given school grade is organized by birth cohort. Therefore, we used school grade as a close proxy for age, with grade 8 corresponding to age 13 and grade 13 corresponding to age 18.

#### 2.3.2 Physical health complaints

Participants were asked how often they had experienced the following health complaints during the past month: “neck and shoulder pain”, “joint and muscle pain”, “headache”, abdominal pain”, “nausea”, and “heart palpitations/chest pain”. The response options were “never”, “a few times”, “many times”, and “daily”. Similar questions have been used in previous studies on health complaints in adolescence (cf., Haugland et al., 2001). We dichotomized each item by collapsing the response options “never” and “a few times” (non-recurrent symptoms, coded 0), and “many times” and “daily” as indicative of recurrent symptoms (coded 1). The dichotomized variables were summarized to create an index of number of recurrent physical health complaints (range 0–6). We chose to focus on recurrent symptoms, as these are more likely to be important for adolescents adjustment and to provide a better indication the severity the trend of PHC, than looking at any occurrence of a given symptom. We also chose this operationalization to better align the results with previous research into PHC in adolescence (Holstein et al., 2021, 2022; Roy et al., 2022).

#### 2.3.3 Physical activity, screen time, and social media use

Physical activity was assessed by an item asking how often they engaged in physical activity that caused them to breathe hard or break a sweat, ranging from 0 (never) to 5 (at least five times a week). Screen time was assessed by a single item assessing daily use of screen outside of school with response options ranging from 0 (no time) to 6 (more than 6 h). Similarly, a single item assessed daily use of social media with response options ranging from 0 (no time) to 5 (more than 3 h). These variables were treated as numeric variables in the analyses.

#### 2.3.4 Municipality level variables

A municipality indicator variable denoting the municipality that each of the adolescents resided in. We also calculated a municipality-year variable that represent the municipality and year of participation. Geographical region (Eastern Norway, Western Norway, Northern Norway, Central Norway, and Southern Norway), and municipality population size (categorized into <5,000, 5000–9,999, 10,000–19,999, 20,000–49,999, 50,000 +) were also included. Information about municipality population size was retrieved from Statistics Norway for the year 2019.

### 2.4 Statistical analyses

We used the multilevel approach detailed by Fairbrother (Fairbrother, 2014) to model repeated cross-sectional data that are comparative as well as longitudinal, where each cross section includes a new sample of respondents drawn from the same set of higher-order units. We used a three-level nesting structure, with individuals (*i*; level 1) nested in municipality-years (*tm*; level 2), nested in municipalities (*m;* level 3). Based on available data, this captures the nested structure of Ungdata, by accounting for dependencies between participants from a given municipality and by municipality years (Schmidt-Catran & Fairbrother, 2016). As we expected that the trends in PHC and its associations with screen time, social media use, and physical activity would be gender dependent, all analyses were gender-stratified but we also tested for gender-interactions.

Our analytical strategy can be divided into a series of steps.

1) In the first set of main models, we modeled a linear trend in PHC for boys and girls separately using the following general equation (Eq.1):

$$y_{itm}=\beta_{o}+\beta_{1}{Time}_{tm}+\beta_{2}{Age}_{itm}+\upsilon_{0m}+u_{0tm}+{є}_{itm}$$

*Y*_*itm*_ represents the number of PHC for adolescent *i* at wave *t* in municipality *m*. *β*_0_ represents the grand intercept across all municipalities, *β*_*1*_ represents the slope of the growth curve, and *β*_*2*_ represents the fixed effects of age represented by a vector of dummy variables. The random intercepts for the municipality- and municipality-year levels are captured by *v*_0*m*_ and *u*_0*tm*_, whereas *e*_*itm*_ represents the individual error term. Random slopes for the time trend were not estimated due to convergence issues, likely due to the relatively little variance on this parameter. We tested for a curvilinear growth curve by inclusion of a quadratic term of the time parameter. Based on model comparisons, a linear function of the time trend provided best fit to data for girls. For the total sample and boys, a quadratic function provided a slightly better fit (Supplementary Table 1). Inspection of the predicted values revealed that the linear and quadratic growth curves were highly similar for the total sample and for boys (Supplementary Fig. 2). Based on the principle of parsimony and to ease the interpretation of the trends, we used a linear function for all models. To test whether number of PHC varied with age, age was represented by a vector of dummy coded variables using a backward difference contrast coding scheme, comparing each age group with the previous age group (e.g., 14-year-olds vs. 13-year-olds, 15-year-olds vs. 14-year-olds). Next, Eq.1 was used on the total sample and expanded by inclusion of two-way and three-way interaction terms to examine if the time trend varied by gender and by age and gender.

2) The next set of main models investigated the influence of co-occurring trends in screen time, social media use, and physical activity (*x*) on the time trends of PHC (*y*). To model the effect of each covariate, we disaggregated the between and within-effects by group mean centering the covariates. First, we calculated the municipalities' means $\left({\bar{x}}_{m}\right)$ and the municipality-year means $\left({\bar{x}}_{tm}\right)$. Next, municipality means were subtracted from the municipality-year means $\left({\bar{x}}_{tm}-{\bar{x}}_{m}\right)$. The resulting longitudinal component $\left({\bar{x}}_{tm}-{\bar{x}}_{m}\right)$ is group mean centered and orthogonal to the municipality mean $\left({\bar{x}}_{m}\right)$, such that both coefficients can be estimated simultaneously and separately (Fairbrother, 2014). Last, we subtracted the municipality-year means from the individual-level effect $\left({\bar{x}}_{itm}-{\bar{x}}_{tm}\right)$. Combined, this gives three coefficients for each covariate (*x*) representing the between-municipality $\left(\beta_{3}\right)$, within-municipality $\left(\beta_{4}\right)$, and the between-individual effects $\left(\beta_{5}\right)$. To ease the interpretation of the intercept, we also grand mean centered the municipality means $\left({\bar{x}}_{m}\right)$ by the population mean $\left(\bar{x}\right)$. Thus, the intercept $\left(\beta_{o}\right)$ represent the number of PHC when the given covariate (*x*) is held at the mean value across all levels of analysis. The group mean centered variables were calculated separately by gender and for the total sample.

We modeled the influence of each covariate separately, before fitting models examining the partial and interactive effects between all pairs of covariates at their respective levels. This was done to further tease apart, for example, whether the effect of screen time on PHC might be explained by time spent on social media use, or if the association would be attenuated or moderated by physical activity levels, as some research suggest there may be such effects (Khan et al., 2021). We finally tested a fully adjusted model entering screen time, social media use, and physical activity simultaneously at all levels. The separate models were estimated using the following general equation (Eq. 2), which were then expanded to include the partial and interactive effects between the covariates:

$$y_{itm}=\beta_{o}+\beta_{1}Time_{tm}+\beta_{2}Age_{itm}+\beta_{3}\left({\bar{x}}_{m}-\bar{x}\right)+\beta_{4}\left({\bar{x}}_{tm}-{\bar{x}}_{m}\right)+\beta_{5}\left(x_{itm}-{\bar{x}}_{tm}\right)+\upsilon_{0m}+u_{0tm}+{є}_{itm}$$

Our first objective was observing the change in the estimate of $\beta_{1}Time$ from the simple growth curve model (Eq.1) to the growth curve as specified above (Eq. 2), as it provides an estimate of how much the slope of the population level time trend in number of PHC changes after accounting for the effects of screen time, social media use, and physical activity across all levels of analysis.

Next, we were most interested in the within-municipality effect, as it provides an estimate of whether municipalities that experience an increase in, for example social media use, over and above the typical level of the municipality, also tend to experience an increase/decrease in number of PHC over and above the typical level of the municipality. From a causal perspective, this longitudinal effect is of particular interest, as it accounts for all unobserved time-invariant differences between municipalities (Bell et al., 2019). Adding *Time* as a Level-1 covariate in Eq.2 also has the beneficial effect that it detrends the within-estimate and accounts for potential simultaneous but unrelated time trends between our potential mechanisms and PHC (Wang & Maxwell, 2015). The within-effect thus provides an estimate of the extent to which the given covariate is associated with the longitudinal variation of number of PHC over and above the general time trend at the municipality level. Last, we examined the partial and interactive effects between all pairs of covariates.

3) To obtain an estimate and test statistic of the contextual effect of the municipality of each covariate, Eq. 2 was adapted by grand mean centering (rather than cluster mean centering) the individual effects (level 1) (Brincks et al., 2017). After this procedure, the between-municipality effect (at level 3) is no longer orthogonal to but adjusted by the individual level effect and translates into a contextual effect of the municipality of each covariate. This contextual effect indicates, for example, whether there is an expected difference in number of PHC between two adolescents who spend an equal amount of time on social media, but lives in municipalities that differ by one unit in social media use at the aggregate level (Brincks et al., 2017). The contextual effect may also be derived from Eq. 2 by subtracting the between-individual estimate from the between-municipality estimate.
4) As sensitivity analyses, we estimated models separately for each physical health complaint, to examine whether the trends varied by symptoms, and whether the between- and within-municipality covariates was associated with some symptoms more than others. We used multilevel linear probability models following the same general equation as specified above, but where the outcome measure is a binary variable denoting the absence (coded 0) or presence (coded 1) of the given symptom. Linear probability models were chosen as we could not consistently estimate multilevel logistic regression models due to convergence issues that we were unable to resolve. The coefficients from the probability models reflect the average marginal effect in terms of proportions of change and thus have the advantage of providing an easy to interpret metric. Simulation studies have shown that LPMs yield reasonable and similar estimates as when using logistic regression models when the outcome variable is neither skewed heavily toward 0 or 1, and when the sample size is not small (both which applies to the current study) (Angrist & Pischke, 2009; Hellevik, 2009). To further assess the potential bias arising from using LPMs, we compared results from multilevel logistic regression models that converged (without screen time, social media use, and physical activity as covariates), with equivalent specified LPMs. The LPMs yielded highly similar predicted probabilities of symptoms across survey years (within 1% points) as estimates obtained from logistic models, lending some support to using LPM on these data.

In all LPMs, a quadratic function of the time trend had better fit to data than a linear function, and was used for the analyses. About 9% had missing data on one or more variables used to create the index of number of PHC. The remaining variables had less than 6% missing. Restricted maximum likelihood (REML) estimation was used in all analysis. At the municipality level, missing data at the outcome measures was addressed with maximum likelihood estimation. At the between-individual level, listwise deletion was used to address missing data. Although techniques for handling missing data in multilevel modelling have improved the last decade, state of the art techniques such as multiple imputation are still not fully equipped to handle three-level multilevel models including interaction terms (Van Buuren, 2018). All analyses were performed with R version 4.2.1 for Mac, using the packages *tidyverse* (Wickham et al., 2019), *lme4* (Bates et al., 2015), and *ggeffects* (Lüdecke, 2018).

## 3 Results

**Fig. 1:**
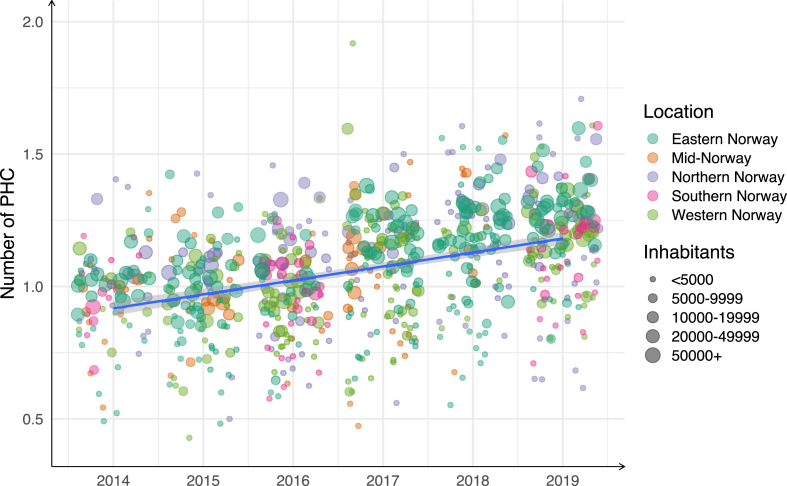
Trends in physical health complaints at the municipality level. *Note.* Each jittered dot represents the municipality mean by survey year. The color and the size of the dot denotes the geographical region and the number of inhabitants in each municipality per 2019. The blue line represents the linear trend at the municipality level.

Means with standard deviations and proportions of focal variables by survey year are shown in Table 1. Descriptively, the number of recurrent PHC and the prevalence for each symptom increased from 2014 to 2019, and more for girls than for boys. In 2019, girls had an average of 1.72 number of PHC, with headache (42.6%), stomach ache (39.9%), and neck and shoulder pain (33.1%) as the most prevalent symptoms. Boys reported an average of 0.76 number of recurrent PHC, with headache (19.5%), neck and shoulder pain (15.9%), and muscle and joint pain (13.0%) as the most prevalent symptoms. Screen time and social media use also increased, whereas a small reduction in physical activity was observed. The overall trend in number of PHC at the municipality level is visualized Fig. 1 and the observed trend for 30 randomly selected municipalities are shown in Supplementary Fig. 2.

**Table 1:** Means and proportions of the main dependent and independent variables across survey years.

	2014	2015	2016	2017	2018	2019
Girls (mean [SD])						
Age	14.92 (1.55)	15.12 (1.60)	14.94 (1.44)	15.15 (1.60)	15.27 (1.66)	15.30 (1.63)
Number of PHC (0–6)	1.37 (1.63)	1.38 (1.64)	1.44 (1.70)	1.62 (1.74)	1.70 (1.77)	1.72 (1.78)
Neck and shoulder pain	28.1%	28.2%	29.8%	33.2%	33.4%	33.1%
Headache	35.4%	35.5%	36.8%	41.3%	42.4%	42.6%
Muscle and joint pain	18.6%	18.5%	19.2%	20.5%	21.0%	20.8%
Nausea	18.5%	18.3%	19.3%	22.2%	23.9%	24.8%
Chest pain	9.8%	11.2%	12.4%	15.1%	16.6%	17.2%
Stomach ache	26.8%	25.9%	26.9%	30.1%	32.4%	33.9%
Screen time (0–6)	3.43 (1.41)	3.46 (1.40)	3.58 (1.39)	3.76 (1.33)	3.82 (1.34)	3.89 (1.28)
Social media (0–5)	3.23 (1.45)	3.26 (1.46)	3.45 (1.41)	3.62 (1.32)	3.59 (1.36)	3.62 (1.31)
Physical activity (0–5)	3.50 (1.18)	3.37 (1.22)	3.41 (1.22)	3.49 (1.16)	3.37 (1.21)	3.43 (1.21)
Individual observations (*n*)	19,552	31,329	29,942	43,710	30,781	51,117

**Boys (mean [SD])**						
Age	14.87 (1.51)	15.08 (1.54)	14.91 (1.41)	15.06 (1.55)	15.19 (1.62)	15.26 (1.59)
Number of PHC (0–6)	0.65 (1.17)	0.62 (1.15)	0.61 (1.14)	0.69 (1.20)	0.76 (1.26)	0.76 (1.26)
Neck and shoulder pain	14.0%	13.5%	13.7%	14.8%	15.9%	15.9%
Headache	16.8%	15.9%	15.5%	18.5%	19.8%	19.5%
Muscle and joint pain	12.5%	11.0%	11.3%	12.0%	12.8%	13.0%
Nausea	7.2%	7.0%	6.5%	8.1%	9.2%	9.1%
Chest pain	5.5%	5.7%	5.4%	6.2%	7.0%	7.0%
Stomach ache	9.0%	8.5%	8.5%	9.8%	10.7%	11.2%
Screen time (0–6)	3.70 (1.47)	3.68 (1.47)	3.75 (1.46)	3.92 (1.40)	4.00 (1.38)	4.05 (1.35)
Social media (0–5)	2.45 (1.50)	2.43 (1.50)	2.49 (1.50)	2.69 (1.47)	2.71 (1.49)	2.73 (1.49)
Physical activity (0–5)	3.76 (1.21)	3.67 (1.25)	3.68 (1.24)	3.74 (1.19)	3.62 (1.24)	3.66 (1.24)
Individual observations (*n*)	19,197	29,332	28,919	42,240	28,293	48,414

Municipalities (*n*)	71	110	122	137	100	129

*Note.* PHC = physical health complaints. The percentages represent the share of respondents reporting recurrent complaints (many times or daily the last month).

### 3.1 Trends in PHC by gender and age

An empty model (not shown) was fitted to derive the interclass correlations (ICC). The ICC for the entire sample was 0.008 at the municipality-year level and 0.003 at the municipality level, suggesting that a total of 1.2% of the variance in PHC was captured by our higher order terms.

A significant linear increase in number of PHC among females (b = 0.08 [95% CI: 0.07, 0.08]) and males (b = 0.03 [95% CI: 0.03, 0.04]) was detected. A one-year higher age was associated with significantly more PHC among females from 13 to 16 years of age. From 16 to 17 years, levels of PHC were slightly lower, and remained stable to 18 years of age. For boys, 14-year-olds had slightly more PHC than 13-year-olds, and levels of PHC remained stable between 14 years and 16 years, before slightly dropping from 17 years of age (see Table 2).

**Table 2:** Gender-stratified trends in number of health complaints by survey year and age.

	Girls			Boys		
	*b*	95% CI	*P*	*b*	95% CI	*p*
Intercept	1.34	1.32, 1.37	<0.001	0.58	0.56, 0.59	<0.001
Time	0.08	0.07, 0.08	<0.001	0.03	0.03, 0.04	<0.001
Age						
14 years (vs. 13 years)	0.33	0.31, 0.35	<0.001	0.10	0.08, 0.11	<0.001
15 years (vs. 14 years)	0.15	0.12, 0.17	<0.001	0.01	0.00, 0.03	0.082
16 years (vs. 15 years)	0.08	0.06, 0.11	<0.001	−0.02	−0.03, 0.00	0.083
17 years (vs. 16 years)	−0.05	−0.08, −0.03	<0.001	−0.07	−0.09, −0.05	<0.001
18 years (vs. 17 years)	−0.01	−0.04, 0.02	0.395	−0.01	−0.04, 0.01	0.274
*Random effects variance*						
Municipality years	0.07			0.03		
Municipality	0.15			0.06		
*Goodness-of-fit*						
−2 Log likelihood	836,954			664,383		
AIC	836,974			664,403		
BIC	837,076			664,506		
*n* adolescents	213,901			206,033		
*n* municipality years	671			671		
*n* municipalities	345			345		

*Note*. 95% CI = 95% confidence interval of b. *b* = unstandardized regression coefficient. Due to the backward difference coding of age, each beta coefficient represents the pairwise difference between each age group and the previous age group (e.g., 14-year-olds compared to 13-year-olds), and the intercepts represent the predicted number of recurrent health complaints when age is set at the gender specific grand mean (Girls = 15.15, Boys = 15.10).

AIC: Akaike Information Criteria. BIC: Bayesian Information Criteria.

A joint model testing the interaction between *time* and *gender* confirmed that the slope of the time trend was significantly steeper among females than males (b = 0.05 [95% CI: 0.04, 0.06], p < 0.001). We also tested a three-way interaction *time, gender,* and *age*, to assess whether differences in the slope of the time trend between boys and girls changed with increasing age. We did not detect a significant three-way interaction (*b* = −0.001, *p* = 0.769) (See Supplementary Fig. 3).

### 3.2 Accounting for the between and within-effects of screen time, social media use, and physical activity

**Fig. 2:**
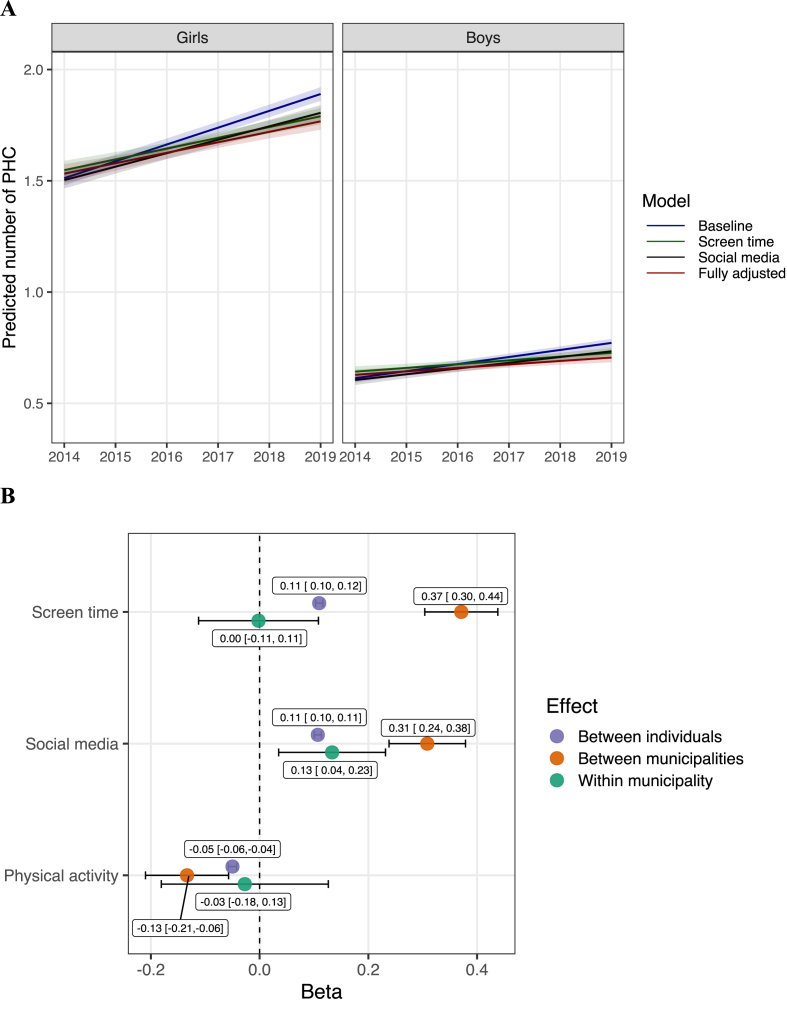
(**A**) Population-level predicted trends of number of physical health complaints across models by gender, and (**B**) regression coefficient plot of the interaction between gender (reference = boys) and screen time, social media use, and physical activity across the between and within levels. *Note.* Panel **A** shows the predicted population level trends in number of health complaints across multilevel regression models shown in Table 3. Each model is also adjusted by age (ref. 16 years). Panel **B** shows the interaction between gender and screen time, social media use, and physical activity across the between and within levels, with *Boys* as the reference group. The error bars represent 95% confidence intervals of *Beta*. Beta = unstandardized regression coefficient. Note that the confidence intervals for the between individual effects are so narrow that the error bars lie within the points.

Accounting for between and within-effects of screen time reduced the predicted slope of the trend with about 0.03 number of PHC for girls and 0.015 number of PHC for boys (using four digits of the estimates; see Table 3). For social media use, the slopes were reduced with 0.015 number of PHC for girls and 0.006 for boys. Physical activity hardly influenced the slope of the time trend. In the fully adjusted models, the slope of the time trend for both genders remained similar as in the model adjusting for screen time. Across all models, the time trend remained statistically significant. The predicted values of the slope of the trend for each model (excluding physical activity which were highly similar to the baseline model) are shown in Fig. 2A.

**Table 3:** Number of health complaints predicted by screen time, social media use, and physical activity.

	Girls			Boys		
	*b*	95% CI	*p*	*b*	95% CI	*p*
**Baseline model**						
Intercept	1.51	1.48, 1.54	<0.001	0.61	0.59, 0.63	<0.001
Time	0.08	0.07, 0.08	<0.001	0.03	0.03, 0.04	<0.001
*Goodness of fit*						
−2 log likelihood	807,328			630,794		
AIC	807,348			630,814		
BIC	807,450			630,916		
**Screen time**						
Intercept	1.55	1.51, 1.59	<0.001	0.64	0.62, 0.67	0.047
Time	0.05	0.04, 0.06	<0.001	0.02	0.01, 0.02	<0.001
Between-individuals	0.21	0.21, 0.22	<0.001	0.10	0.10, 0.11	<0.001
Between-municipalities	0.52	0.42, 0.62	<0.001	0.22	0.17, 0.26	<0.001
Within-municipality change	0.25	0.15, 0.35	<0.001	0.18	0.12, 0.25	<0.001
*Goodness of fit*						
−2 log likelihood	801,480			627,889		
AIC	801,506			627,915		
BIC	801,639			628,047		
**Social media use**						
Intercept	1.50	1.47, 1.54	<0.001	0.60	0.58, 0.63	0.077
Time	0.06	0.05, 0.07	<0.001	0.03	0.02, 0.03	<0.001
Between-individuals	0.20	0.20, 0.21	<0.001	0.10	0.09, 0.10	<0.001
Between-municipalities	0.43	0.33, 0.53	<0.001	0.18	0.12, 0.24	<0.001
Within-municipality change	0.19	0.09, 0.29	<0.001	0.10	0.04, 0.15	0.001
*Goodness of fit*						
−2 log likelihood	801,823			628,048		
AIC	801,849			628,074		
BIC	801,982			628,207		
**Physical activity**						
Intercept	1.51	1.48, 1.54	<0.001	0.61	0.59, 0.63	<0.001
Time	0.08	0.07, 0.08	<0.001	0.03	0.03, 0.04	<0.001
Between-individuals	−0.06	−0.07, −0.06	<0.001	−0.01	−0.02, −0.01	<0.001
Between-municipalities	−0.26	−0.38, −0.14	<0.001	−0.07	−0.13, 0.00	0.042
Within-municipality change	−0.07	−0.20, 0.05	0.243	−0.07	−0.16, 0.02	0.129
*Goodness of fit*						
−2 log likelihood	806,913			630,764		
AIC	806,939			630,791		
BIC	807,072			630,923		

*Note.* Separate models examining the associations between screen time, social media use, and physical activity across all levels of analysis. Each model is also adjusted by age (ref. 16 years). *b* = unstandardized regression coefficient 95% CI = 95% confidence interval of *b. N*_*Girls*_: 206,431, *N*_Boys_: 196,395, *N*_Municipality years_: 669, *N*_Municipalities_: 345 (across all models). Goodness of fit measures are rounded to the nearest whole number. Random effects variances for all models: Baseline model (Girls/Boys): municipality -year = 0.004, municipality = 0.022/municipality-year = 0.001, municipality year = 0.003. Screen time model (Girls/Boys): municipality -year = 0.004, municipality = 0.010/municipality-year = 0.001, municipality = 0.002 Social media models (Girls/Boys): municipality -year = 0.005, municipality = 0.023/municipality-year = 0.001, municipality = 0.003 Physical activity models (Girls/Boys): municipality -year = 0.005, municipality = 0.021/municipality-year = 0.001, municipality = 0.003 AIC: Akaike Information Criteria.

BIC: Bayesian Information Criteria.

**Fig. 3:**
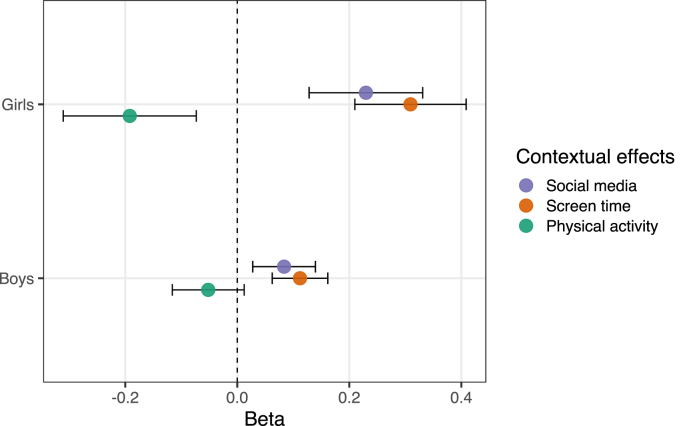
The contextual effect of municipality levels of social media, screen time, and physical activity. *Note.* The separate municipality level contextual effects of social media use, screen time, and physical activity for boys and girls. Error bars represent the 95% confidence interval of beta. Beta = unstandardized regression coefficient. The contextual effect refers to how much the municipality level of social media, screen time, and physical activity is associated with adolescents PHC independent of the individual level association.

### 3.3 The between and within-effects of screen time, social media use, and physical activity

For screen time and social media use, coefficients across all levels were positive and statistically significant. At the between-municipality level for girls, a one-unit higher level of screen time was associated with 0.52 higher number of PHC. The significant within-municipality effect of screentime further suggested that in years when screen time *increased* with one unit from the typical level of a given municipality, there was a predicted *increase* in number of PHC by 0.25. The same pattern was observed for boys, although the strength of the associations was weaker, particularly on the between-municipality level (*b*_between-municipalities_ = 0.22; *b*_within-municipality_ = 0.18). Interaction analyses confirmed that higher levels of social media use was more strongly and positively associated with PHC among girls than boys across all levels of analysis. For screen time and physical activity, only the gender interaction with the between-effects were statistically significant (see Fig. 2B).

In analyses investigating the partial and interactive effects between the covariates, the effect of screen time (across all levels) only slightly attenuated when adjusted by social media use (for girls and boys). Thus, although some of the association between screen time and PHC appear to be explained by social media use, both screen time and social media use had independent associations with PHC. We also detected significant but very weak interaction effects between screen time and social media use at the between-individual level for girls and boys. Given how weak these effects were, we do not consider them to be of any practical importance.

Adjusting for physical activity hardly influenced the associations between screen time and social media use on PHC (across all levels of analyses). Moderation analyses between screen time and physical activity, and social media use and physical activity, did not detect any effect modifications between these variables, with the exception of a significant interaction between screen time and physical activity at the between-municipality level for girls (for full results, see Supplementary Tables 2–7, and Supplementary Fig. 4).

### 3.4 The contextual effects of screen time, social media use, and physical activity

The contextual effects of each covariate at the municipality level are illustrated in Fig. 3.

For girls, a one-unit higher municipality level of screen time and social media use were associated with about 0.2–0.3 higher number of PHC independent of adolescent girls’ individual level use. The contextual effect of physical activity was negative, suggesting that a one-unit higher municipality level of physical activity was associated with about 0.2 lower number of PHC, over and above the individual level of physical activity. The magnitude of the contextual effects for boys were smaller, and the contextual effect of physical activity was not statistically significantly different from zero.

### 3.5 Trends in specific PHC symptoms

Findings from these analyses generally mimic the results obtained from using the index of total number of PHC as the outcome but provide some additional insights. For both girls and boys, a significant increase was detected for all symptoms, although the shape of the trend varied somewhat between the symptoms. As for number of health complaints, the trend for each symptom was stronger for girls than boys. For girls, the most prominent increase was in the prevalence of headache, stomach ache, and chest pain. For boys, the trends was observably more similar across the symptoms (see Supplementary Fig. 4 for the slopes of the time trends).

**Fig. 4:**
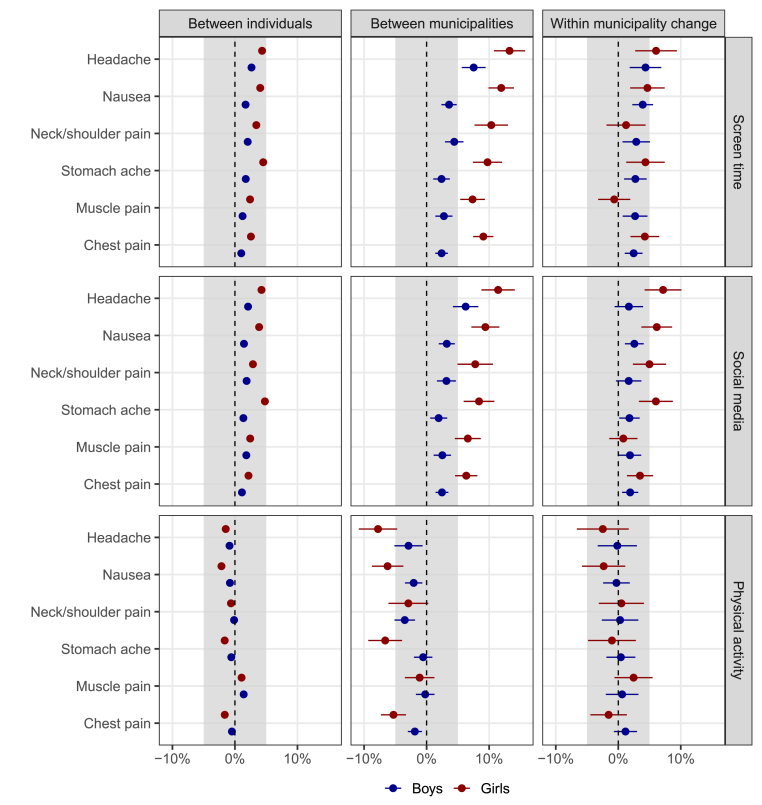
Linear probability models of the between and within associations between physical activity, screen time, and social media use on physical health complaints symptoms. *Note.* Results from linear probability models of the between-individuals (left column), between-municipalities (mid column) and within-municipality (right column) associations between screen time (top row), and social media use (mid row), and physical activity (bottom row), on each physical health complaint symptom. Each dot represents the predicted probability of experiencing recurrent symptoms by a one-unit higher physical activity, screen time, and social media use across levels. Error bars represent 95% confidence intervals. Estimates with error bars crossing the dotted vertical line were not statistically significant at p < 0.05. Note that the confidence intervals for the between individual effects are so narrow that the error bars lie within the points.

A one-unit higher screen time and social media use on the municipality level was associated with about 7%–10% increased municipality prevalence across the symptoms for girls and about 2–6% for boys. The within-municipality effect was particularly prominent for social media use for girls, and for headache, nausea, neck/shoulder pain and stomach ache, where a one unit increase in municipality level of social media use was associated with about a 5–7% increased prevalence of these symptoms at the municipality level. The within-municipality effect of social media use for boys was, however, small, and mostly not statistically significant across the symptoms. Physical activity was not longitudinally associated with municipality level prevalence (see Fig. 4 for details).

## 4 Discussion

This nationwide study of Norwegian adolescents found that physical health complaints (PHC) increased from 2014 to 2019, particularly among adolescent girls. Screen time and social media use attenuated a moderate part of the time trends in PHC. We further detected significant between-municipalities and within-municipality (longitudinal) associations of screen time and social media on adolescents’ PHC. Physical activity, on the other hand, was only associated with PHC at the between individual and between municipality level. Overall, the results indicate that trends in screen time and social media use are intertwined with the rising trends in PHC among adolescents, and more so for girls than boys. Our findings also provide some evidence that the prevalence of PHC may be linked to changing processes in the youth culture set into motion by higher screen time and social media use at the group level.

The model-based increase from 2014 to 2019 was approximately 0.8 number of PHC for girls and 0.3 number of PHC for boys. For girls, all symptoms, except muscle and joint pain, had a predicted increased prevalence of about 5–8%. These results are noteworthy, given the relatively short time frame of six years. These results align with findings from other Western high-income countries and the common conclusion that the trends are more prominent among girls than boys (Cosma et al., 2020; Ottova-Jordan et al., 2015; Potrebny et al., 2019).

Previous work suggests that the trends in mental health problems and PHC such as back pain are stronger among older adolescent girls than younger girls and boys (Potrebny et al., 2019; Roy et al., 2022). We did not detect this age*gender pattern, as the trend was stable across all age groups within each gender. This may stem from differences in periods and contexts investigated or other methodological features of existing work. However, it may also point to different age and gender-specific trends in PHC compared to measures of anxiety and depression, or that such interactions only exist for specific PHC such as back pain, which were not further explored in the current study. Future studies are needed to better address this.

It has been suggested that PHC increases from early to late adolescence, likely due to the pubertal development in this period (LeResche et al., 2005; Swain et al., 2014). Our findings suggest that this holds true for girls from 13 to 16 years of age. For boys, levels of PHC were more stable across age groups, with the only observed increase from 13 to 14 years of age. However, according to our findings, levels of PHC do not seem to get higher at older ages, and thus nuances the view that PHC continuously increase during adolescence. As detailing the developmental trajectory of PHC during adolescence was not the main aim of this study, we urge that future studies, ideally using longitudinal designs, may better answer such questions and potentially corroborate our findings. There may also be different developmental trajectories across different symptoms, a question not explored in the present study.

Accounting for screen time across all levels of analysis attenuated the slope of the time trend with about 0.03 number of PHC (per year) for girls and 0.015 for boys. Social media use attenuated the time trend by about 0.015 for girls and 0.006 for boys. These results suggest that in the hypothetical scenario that screen time and social media use had remained stable across the period investigated, the trend in PHC would have been less prominent. A previous study found that screen time only very slightly attenuated the slope of the trend in back pain from 2002 to 2014 (Roman-Juan et al., 2022). However, by only providing aggregated estimates across 27 European countries and for an earlier period, it is hard to compare their results to ours.

Compared to studies examining other hypothesized mechanisms, such as school (work) stress (Cosma et al., 2020; Högberg et al., 2020), body dissatisfaction and cannabis use (von Soest & Wichstrøm, 2014), and changing parent-adolescent relationships (De Looze et al., 2020), the attenuating effect of screen time and social media use detected in this study was stronger. For example, a recent study found that school stress only weakly attenuated the time trend in psychosomatic symptoms in Sweden (Högberg et al., 2020). Still, we caution against drawing too strong inferences based on comparisons with studies from, in part, other periods, contexts, using different methods, and measurements. Indeed, most previous work has focused on internalizing or psychosomatic problems (a scale combining PHC and mental health problems). Although internalizing problems and PHC tend to correlate strongly (Dey et al., 2015), they do not necessarily associate equally with screen time and social media use. Moreover, our findings highlight that other factors are also important, as we could not fully explain the rising trend in PHC by screen time and social media use.

Screen time and social media use were consistently associated with PHC across all levels of analysis and more strongly for girls than boys. Although some of the effect of screen time attenuated when considered jointly with social media use, our results suggest that screen time and social media use also have independent associations with PHC. Several individual-level mechanisms of these associations have been proposed, such as ergonomic aspects of screen-based activities (Torsheim et al., 2010), and stress through social comparison, information overload, and reduced face-to-face interactions (Wolfers & Utz, 2022). However, as between effects may be confounded by several unmeasured variables, we do not put too much emphasis on the estimates at the between-level.

More noteworthy, we detected significant within-municipality effects of screen time and social media use on PHC. The within associations suggests that the prevalence of PHC at the municipality level rose in tandem with a municipality-level shift toward higher screen time and social media use. In addition to the individual-level mechanisms noted above, this result may also be driven by a shift in how adolescents socialize. The expansion of the smartphone may have led to online interaction gradually substituting in-person socialization (Twenge et al., 2021). As social interaction is a group-level phenomenon, this may influence adolescents independent of their individual level of screen time and social media use. Such a contextual effect was also detected in the present study, whereby particularly for girls, more screen time and social media use at the municipality level was associated with higher levels of PHC independent of adolescent girls’ individual use. Online interaction has been linked to less emotional closeness than in-person socialization, and a recent study found evidence that more digital media use (at the school level) was predictive of higher levels of loneliness (Twenge et al., 2021). Our findings complement these results and point to PHC as another outcome of higher group levels of screen time and social media use.

A group-level shift toward higher social media use may also have led to a youth culture more focused on social comparison and pressured to succeed in multiple areas of life. These social pressures may have yielded a youth culture that is more stressed than previous generations, which could also manifest through higher levels of PHC (Alfven et al., 2008; Wiklund et al., 2012). Although we cannot determine the processes by which digital media use is linked to PHC, our results indicate that the expansion of social media is more strongly associated with PHC in girls than boys.

### 4.1 Strengths and limitations

A strength of the current study was the nationwide sample of adolescents based on yearly repeated survey data with a high response rate across each survey year. This provides some confidence that the results reasonably well describe population-level trends in PHC in Norway from 2014 to 2019. Another key strength was the disaggregation of the between- and within-municipality effects. The within-municipality effect accounts for all time-invariant differences at the municipality level and thus provides a more compelling argument of longitudinal associations between screen time and social media use on PHC at the municipality level than between comparisons. The large sample of higher-order units is an additional strength as it provides more statistical power than common within similar multilevel research, often drawing on fewer higher-order units such as countries (Bryan & Jenkins, 2016).

Some limitations should be acknowledged. First, although motivated by an interest in identifying the determinants of trends in adolescent well-being, observational research as the current cannot ascertain that the reported associations between screen time and social media use on PHC represent causal effects. There may be other unmeasured (time-varying) variables that could explain our results. Notably, we cannot exclude the possibility that the co-relation between trends in screen time, social media use and PHC stem from changing trends in a common cause factor in which we have not been able to control for. Moreover, we cannot exclude the possibility of reversed causality, whereby high levels of PHC lead to more screen time and social media use.

Another limitation pertains to our measures of screen time and social media use. These only capture time spent on these activities and do not separate between different types of activities adolescents may engage in. For example, recent work has highlighted that passive and active digital media use may have unique associations with adolescents’ well-being (Tang et al., 2021). Thus, more multifaceted measures could have provided nuances to the findings reported in this study. Another potential limitation was that our measure of screen time was limited to time spent on screen-based devices outside school hours and hence do not capture the potential effect of using screens at school.

Our study is also limited by the fact that single-item measures, as our measures of screen time, social media use, and physical activity, have unknown reliability, and previous work has suggested that respondents tend to overestimate time spent on such activities (e.g., Johannes et al., 2021). However, research has also shown that single-item measures may be as valid and reliable as their multi-item counterparts (Allen et al., 2022). Moreover, although more objective measures are preferable, such measures are often difficult if not impossible to use in large scale population studies as the current.

Finally, we used multilevel linear probability models (LPMs) instead of multilevel logistic regression analyses in our secondary analyses, as the multilevel logistic regression analyses yielded convergence issues. LPMs have the benefit of providing an easy to interpret metric and have been shown to work well under several circumstances (Hellevik, 2009). Robustness analyses on simpler models that converged also showed high similarity between multilevel logistic regression models and LPMs on the current data. However, we cannot exclude the possibility that the LPMs may have induced some imprecisions in our secondary results.

## 5 Conclusion

From 2014 to 2019, PHC increased among Norwegian adolescents, particularly among girls. During this period, there has also been an increase in time spent on screen-based devices, including social media use. Accounting for these conjoint trends across the individual and municipality level of analysis attenuated a moderate part of the slope of the trend in PHC for girls and to a lesser extent for boys. The within-municipality effect of screen time and social media use provides some evidence that the population-level trends in PHC in Norway may be partly fueled by a population-level shift toward higher screen time and social media use. Although we cannot establish the mechanisms that underlie this pattern, it is plausible that a combination of individual-level mechanisms and the contextual effects of growing up in a society where the use of digital media technology is high, jointly contribute to the rising trends in PHC. In parallel, the digital media revolution certainly has its positive features as well. Thus, there is a need for future studies that better can address how various aspects of adolescents' digital life relate to both positive and negative aspects of their well-being. Last, it is important to stress that our results highlight that other aspects of adolescents’ lives are important, as screen time and social media use did not fully account for the increasing trend of PHC. Indeed, the causes of PHC in adolescence is likely multifaceted. Thus, we urge future work to continue considering other causes of these trends besides physical activity, screen time, and social media use.

## Ethical statement

Hereby, I, Sondre Aasen Nilsen, consciously assure that for the manuscript “*Trends in physical health complaints among adolescents from 2014 – 2019: Considering screen time, social media use, and physical activity”,* the following is fulfilled.

1) This material is the authors' own original work, which has not been previously published elsewhere.
2) The paper is not currently being considered for publication elsewhere.
3) The paper reflects the authors' own research and analysis in a truthful and complete manner.
4) The paper properly credits the meaningful contributions of co-authors and co-researchers.
5) The results are appropriately placed in the context of prior and existing research.
6) All sources used are properly disclosed (correct citation).
7) All authors have been personally and actively involved in substantial work leading to the paper, and will take public responsibility for its content.

Date: 05.01.2023.

## Author statement

**Sondre Aasen Nilsen**: Conceptualization, Methodology, Software, Formal analysis, Writing - Original Draft, Visualization.

**Kjell Morten Stormark:** Conceptualization, Writing - Review & Editing, Project administration.

**Ove Heradstveit**: Conceptualization, Writing - Review & Editing.

**Kyrre Breivik:** Conceptualization, Methodology, Formal analysis, Writing - Review & Editing.

## Declaration of competing interest

None.

## Data Availability

Data are available for research on application (ungdata.no).
