# Supplementary material for: Trends in physical health complaints among adolescents from 2014 – 2019: Considering screen time, social media use, and physical activity
Source: SSM Popul Health. 2023 Apr 11;22:101394. doi: 10.1016/j.ssmph.2023.101394 (PMC10126924; doi:10.1016/j.ssmph.2023.101394)
Supplement: Multimedia component 1 [file mmc1.docx]

**Supplementary materials**

| **Supplementary Table 1.** Model fit of nested models comparing a linear function of the time trend with a quadratic function of the time trend. | | | | | |
| --- | --- | --- | --- | --- | --- |
|  | AIC | BIC | Log Likelihood | Chi-square | p value |
| **Total sample** |  |  |  |  |  |
| Linear | 1527393 | 1527513 | -763685 |  |  |
| Quadratic | 1527391 | 1527522 | -763683 | 3.9213 | 0.048 |
|  |  |  |  |  |  |
| **Girls** |  |  |  |  |  |
| Linear | 836921 | 837024 | -418451 |  |  |
| Quadratic | 836923 | 837036 | -418451 | 0.206 | 0.650 |
|  |  |  |  |  |  |
| **Boys** |  |  |  |  |  |
| Linear | 664345 | 664448 | -332163 |  |  |
| Quadratic | 664323 | 664436 | -332151 | 24.126 | < 0.001 |
| *Note.* AIC: Akaike Information Criteria, BIC: Bayesian Information Criteria Estimation: Maximum Likelihood | | | | | |

**Supplementary Table 2**. Adjusted and interactive effects of screen time and physical activity on PHC among girls

|  | Adjusted model  ***b* (95% CI)** |  | Interaction model ***b* (95% CI)** | |
| --- | --- | --- | --- | --- |
| Intercept | 1.55 (1.50,1.59)** | | 1.56 (1.51,1.60)** | |
| Time | 0.05 (0.04, 0.06)** | | 0.05 (0.04, 0.06)** | |
| **Screen time** |  |  |  |  |
| Between-individuals | 0.21 (0.20, 0.21)** | | 0.21 (0.20, 0.21)** | |
| Between-municipalities | 0.51 (0.40, 0.61)** | | 0.51 (0.40, 0.62)** | |
| Within-municipality change | 0.25 (0.14, 0.35)** | | 0.26 (0.15, 0.36)** | |
| **Physical activity** |  |  |  |  |
| Between-individuals | -0.03 (-0.03, -0.02)** | | -0.03 (-0.03, -0.02)** | |
| Between-municipalities | -0.05 (-0.17, 0.07) |  | -0.05 (-0.17, 0.06) | |
| Within-municipality change | -0.02 (-0.14, 0.11) |  | -0.02 (-0.14, 0.11) | |
| **Screen time*physical activity** |  |  |  |  |
| Between-individuals |  |  | 0.00 (0.00, 0.01) | |
| Between-municipalities |  |  | 0.54 (0.11, 0.96)* | |
| Within-municipality change |  |  | 0.02 (-0.54, 0.58) | |
| *Random effect variances* |  |  |  |  |
| Municipality year | 0.00384 |  | 0.00385 |  |
| Municipality | 0.01478 |  | 0.01431 |  |
| *Goodness of fit* |  |  |  |  |
| -2 log likelihood | 801,422 |  | 801,428 |  |
| AIC | 801,454 |  | 801,466 |  |
| BIC | 801,618 |  | 801,661 |  |

*Note.* Adjusted and interactive effects of screen time and physical activity on number of physical health complaints (PHC). Also adjusted for age (reference group = 16 years).

AIC: Akaike Information Criteria

BIC: Bayesian Information Criteria

**Supplementary Table 3**. Adjusted and interactive effects of social media use and physical activity on PHC among girls

|  | Adjusted model  ***b* (95% CI)** |  | Interaction model ***b* (95% CI)** | |  |
| --- | --- | --- | --- | --- | --- |
|  |  |  |  |  |  |
| Intercept | 1.50 (1.46, 1.54)** | | 1.50 (1.46, 1.54)** | |  |
| Time | 0.06 ( 0.05, 0.07)** | | 0.06 ( 0.05, 0.07)** | |  |
| **Social media** |  |  |  |  |  |
| Between-individuals | 0.20 ( 0.20, 0.21) ** | | 0.20 ( 0.197, 0.21) ** | | |
| Between-municipalities | 0.40 ( 0.30, 0.51)** | | 0.40 ( 0.30, 0.51)** | |  |
| Within-municipality change | 0.18 (0.08, 0.28)** | | 0.18 (0.08, 0.28) | |  |
| **Physical activity** |  |  |  |  |  |
| Between-individuals | -0.06 ( -0.06, -0.05)** | | -0.06 ( -0.06, -0.05)** | | |
| Between-municipalities | -0.15 (-0.27, -0.04)* | | -0.15 (-0.26, -0.03)* | | |
| Within-municipality change | -0.07 (-0.19, 0.05) | | -0.06 (-0.18, 0.07) | |  |
| **Social media*physical activity** |  |  |  |  |  |
| Between-individuals |  |  | 0.004 (<-0.0001, 0.0082) | | |
| Between-municipalities |  |  | -0.0004 (-0.50, 0.49) | | |
| Within-municipality change |  |  | -0.39 (-1.04 , 0.268) | |  |
| *Random effect variances* |  |  |  |  |  |
| Municipality year | 0.0040 |  | 0.0040 |  |  |
| Municipality | 0.0164 |  | 0.0164 |  |  |
| *Goodness of fit* |  |  |  |  |  |
| -2 log likelihood | 801,508 |  | 801,515 |  |  |
| AIC | 801,540 |  | 801,553 |  |  |
| BIC | 801,703 |  | 801,747 |  |  |

*Note.* Adjusted and interactive effects of social media use and physical activity on number of physical health complaints (PHC). Also adjusted for age (reference group = 16 years).

AIC: Akaike Information Criteria

BIC: Bayesian Information Criteria

**Supplementary Table 4**. Adjusted and interactive effects of screen time and physical activity on PHC among boys

|  | Adjusted model  ***b* (95% CI)** | | Interaction model  ***b* (95% CI)** | |
| --- | --- | --- | --- | --- |
| Intercept | 0.64 (0.62, 0.67)** | | 0.65 (0.62, 0.67)** | |
| Time | 0.02 (0.01, 0.02)** | | 0.02 (0.01, 0.02)** | |
| **Screen time** |  |  |  |  |
| Between-individuals | 0.10 (0.10, 0.11)** | | 0.10 (0.10, 0.11)** | |
| Between-municipalities | 0.22 (0.17, 0.27)** | | 0.22 (0.17, 0.28)** | |
| Within-municipality change | 0.18 (0.11, 0.25)** | | 0.18 (0.11, 0.25)** | |
| **Physical activity** |  |  |  |  |
| Between-individuals | 0.01 (0.00, 0.01)* | | 0.01 (0.00, 0.01)* | |
| Between-municipalities | 0.01 (-0.05, 0.07) | | 0.01 (-0.05, 0.08) | |
| Within-municipality change | -0.04 (-0.13, 0.05) | | -0.04 (-0.13, 0.05) | |
| **Screen time*physical activity** |  |  |  |  |
| Between-individuals |  |  | 0.00 (0.00, 0.00) | |
| Between-municipalities |  |  | 0.15 (-0.10, 0.40) | |
| Within-municipality change |  |  | 0.00 (-0.47, 0.47) | |
| *Random effect variances* |  |  |  |  |
| Municipality year | 0.00106 |  | 0.00105 |  |
| Municipality | 0.00202 |  | 0.00202 |  |
| *Goodness of fit* |  |  |  |  |
| -2 log likelihood | 627,900 |  | 627,911 |  |
| AIC | 627,932 |  | 627,949 |  |
| BIC | 628,095 |  | 628,143 |  |

*Note.* Adjusted and interactive effects of screen time and physical activity on number of physical health complaints (PHC). Also adjusted for age (reference group = 16 years).

AIC: Akaike Information Criteria

BIC: Bayesian Information Criteria

**Supplementary Table 5**. Adjusted and interactive effects of social media use and physical activity on PHC among boys

|  | Adjusted model  ***b* (95% CI)** | | Interaction model  ***b* (95% CI)** | |
| --- | --- | --- | --- | --- |
| Intercept | 0.60 (0.58, 0.63)** | | 0.60 (0.58, 0.62)** | |
| Time | 0.02 (0.02, 0.03)** | | 0.02 (0.02, 0.03)** | |
| **Social media** |  |  |  |  |
| Between-individuals | 0.10 (0.09, 0.10)** | | 0.10 (0.09, 0.10)** | |
| Between-municipalities | 0.18 (0.12, 0.23)** | | 0.17 (0.12, 0.23)** | |
| Within-municipality change | 0.10 (0.04, 0.15)** | | 0.10 (0.04, 0.16)** | |
| **Physical activity** |  |  |  |  |
| Between-individuals | -0.02 (-0.03, -0.02)** | | -0.02 (-0.03, -0.02)** | |
| Between-municipalities | -0.05 (-0.11, 0.02) | | -0.04 (-0.10, 0.02) | |
| Within-municipality change | -0.08 (-0.17, 0.00) | | -0.09 (-0.17, 0.00) | |
| **Social media*physical activity** |  |  |  |  |
| Between-individuals |  |  | 0.00 (0.00, 0.00) | |
| Between-municipalities |  |  | -0.13 (-0.41, 0.14) | |
| Within-municipality change |  |  | -0.39 (-0.86, 0.09) | |
| *Random effect variances* |  |  |  |  |
| Municipality year | 0.00128 |  | 0.00123 |  |
| Municipality | 0.00250 |  | 0.00252 |  |
| *Goodness of fit* |  |  |  |  |
| -2 log likelihood | 627,946 |  | 627,954 |  |
| AIC | 627,978 |  | 627,992 |  |
| BIC | 628,141 |  | 628,186 |  |

*Note.* Adjusted and interactive effects of social media use and physical activity on number of physical health complaints (PHC). Also adjusted for age (reference group = 16 years).

AIC: Akaike Information Criteria

BIC: Bayesian Information Criteria

**Supplementary Table 6**. Adjusted and interactive effects of screen time and social media use on PHC among girls

|  | Adjusted model  ***b* (95% CI)** |  | Interaction model  ***b* (95% CI)** | |
| --- | --- | --- | --- | --- |
| Intercept | 1.54 (1.49, 1.58)** | | 1.52 (1.47, 1.56)** | |
| Time | 0.05 (0.03, 0.06)** | | 0.05 (0.04, 0.06)** | |
| **Screen time** |  |  |  |  |
| Between-individuals | 0.15 (0.14, 0.15)** | | 0.15 (0.14, 0.16)** | |
| Between-municipalities | 0.44 (0.29, 0.60)** | | 0.47 (0.31, 0.62)** | |
| Within-municipality change | 0.22 (0.09, 0.34)** | | 0.22 (0.09, 0.34)** | |
| **Social media** |  |  |  |  |
| Between-individuals | 0.14 (0.13, 0.14)** | | 0.14 (0.13, 0.14)** | |
| Between-municipalities | 0.11 (-0.04, 0.27) |  | 0.10 (-0.05, 0.26) | |
| Within-municipality change | 0.08 (-0.05, 0.20) |  | 0.09 (-0.03, 0.21) | |
| **Screen time*social media** |  |  |  |  |
| Between-individuals |  |  | 0.02 (0.02, 0.02)** | |
| Between-municipalities |  |  | -0.30 (-0.63, 0.03) | |
| Within-municipality change |  |  | 0.27 (-0.07, 0.61) | |
| *Random effect variances* |  |  |  |  |
| Municipality year | 0.00362 |  | 0.00366 |  |
| Municipality | 0.01497 |  | 0.01457 |  |
| *Goodness of fit* |  |  |  |  |
| -2 log likelihood | 799,599 |  | 799,493 |  |
| AIC | 799,631 |  | 799,531 |  |
| BIC | 799,794 |  | 799,725 |  |

*Note.* Adjusted and interactive effects of screen time and social media use on number of physical health complaints (PHC). Also adjusted for age (reference group = 16 years).

AIC: Akaike Information Criteria

BIC: Bayesian Information Criteria

**Supplementary Table 7**. Adjusted and interactive effects of screen time and social media use on PHC among boys

|  | Adjusted model  ***b* (95% CI)** |  | Interaction model  ***b* (95% CI)** |  |
| --- | --- | --- | --- | --- |
| Intercept | 0.63 (0.61, 0.65)** | | 0.62 (0.59, 0.64)** | |
| Time | 0.02 (0.01, 0.02)** | | 0.02 (0.01, 0.02)** | |
| **Screen time** |  |  |  |  |
| Between-individuals | 0.09 (0.08, 0.09)** | | 0.09 (0.08, 0.09)** | |
| Between-municipalities | 0.18 (0.13, 0.24)** | | 0.19 (0.13, 0.25)** | |
| Within-municipality change | 0.17 (0.09, 0.25)** | | 0.17 (0.10, 0.25)** | |
| **Social media** |  |  |  |  |
| Between-individuals | 0.08 (0.08, 0.08)** | | 0.07 (0.07, 0.08)** | |
| Between-municipalities | 0.09 (0.03, 0.15)** | | 0.09 (0.02, 0.15)** | |
| Within-municipality change | 0.05 (-0.01, 0.11) | | 0.05 (-0.01, 0.11) | |
| **Screen time*social media** |  |  |  |  |
| Between-individuals |  |  | 0.01 (0.01, 0.02)** | |
| Between-municipalities |  |  | 0.00 (-0.22, 0.22) | |
| Within-municipality change |  |  | 0.19 (-0.09, 0.46) |  |
| *Random effect variances* |  |  |  |  |
| Municipality year | 0.000956 |  | 0.000908 |  |
| Municipality | 0.002021 |  | 0.002057 |  |
| *Goodness of fit* |  |  |  |  |
| -2 log likelihood | 626,144 |  | 626,020 |  |
| AIC | 626,176 |  | 626,058 |  |
| BIC | 626,339 |  | 626,251 |  |

*Note.* Adjusted and interactive effects of screen time and social media use on number of physical health complaints (PHC). Also adjusted for age (reference group = 16 years).

AIC: Akaike Information Criteria

BIC: Bayesian Information Criteria

**Supplementary Figure 1**. Predicted slopes of the time trend in number of physical health complaints for the total sample and for boys. Comparing linear and quadratic functions of the time trend.

Note. This figures shows the predicted slopes of the time trend when using a linear function (lm) and a quadratic function of the time trend for the total sample and for boys, where model comparisons suggested that a quadratic function of the time trend had better fit to data than a linear function of the time trend.

**Supplementary Figure 2**. Trends in physical health complaints at 30 randomly selected municipalities.


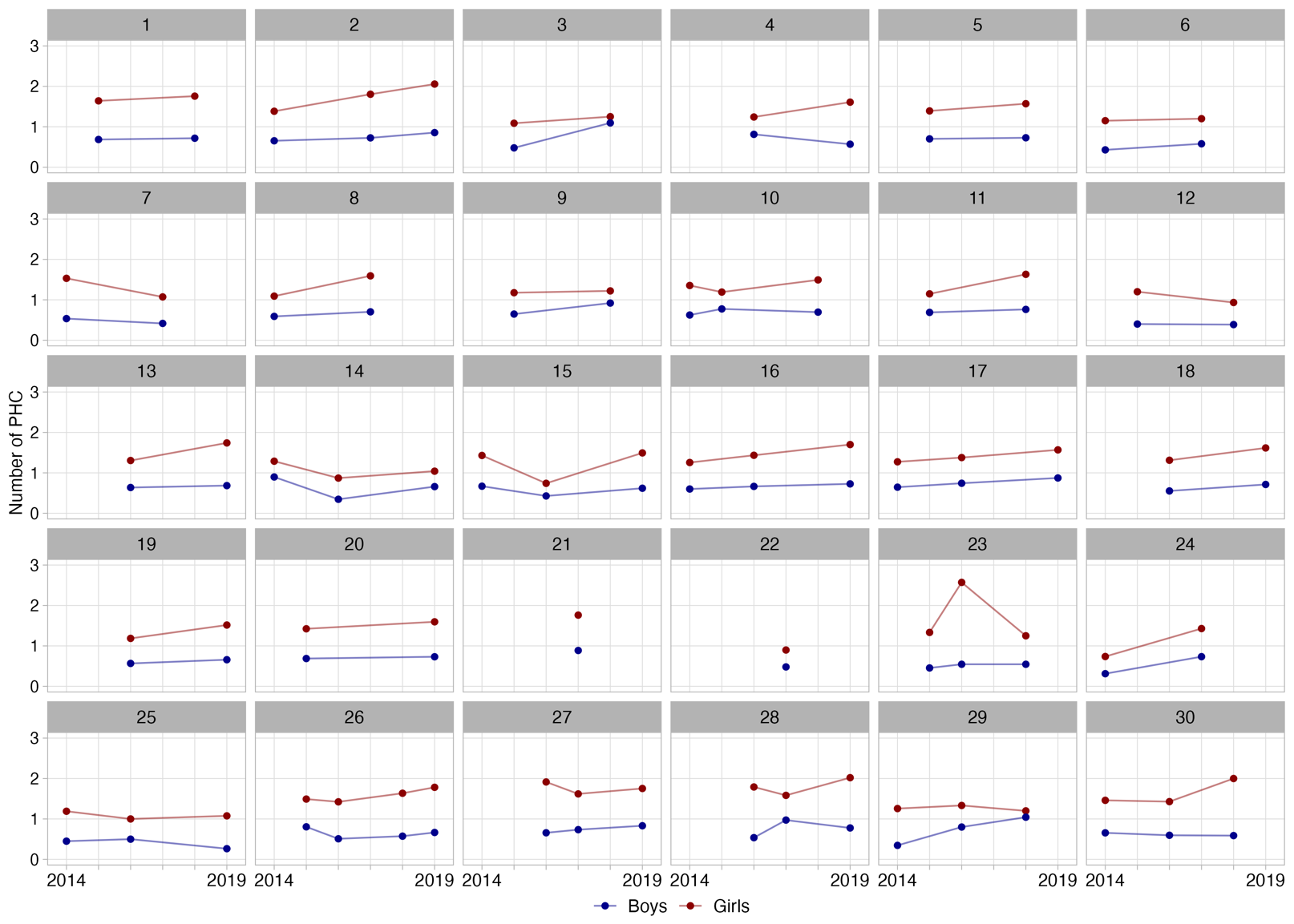


*Note.* Trends in number of physical health complaints by gender for 30 random selected municipalities.

**Supplementary Figure 3.** Three-way interaction between the time trend in physical health complaints, gender, and age.

*Note.* Predicted time trends in number of PHS by age and gender from multilevel models. The light red and light blue shaded areas represent 95 % confidence intervals.
T1 = 2014, T6 = 2019.

**Supplementary Figure 4**. The interaction between screen time and physical activity at the between-municipality level for girls

*Note.* This figure shows the interaction effect between screen time and physical activity on number of PHC at the between-municipalities level for girls. The lines represents the predicted slopes of the association between screen time and PHC at different levels of physical activity at the municipality level(-0.5 = 0.5 units below the mean, 0 = at the mean, 0.5 = 0.5 units above the mean). Adjusted by age (reference group = 16 years)

**Supplementary Figure 5**. Growth curves from linear probability models for each health complaint.


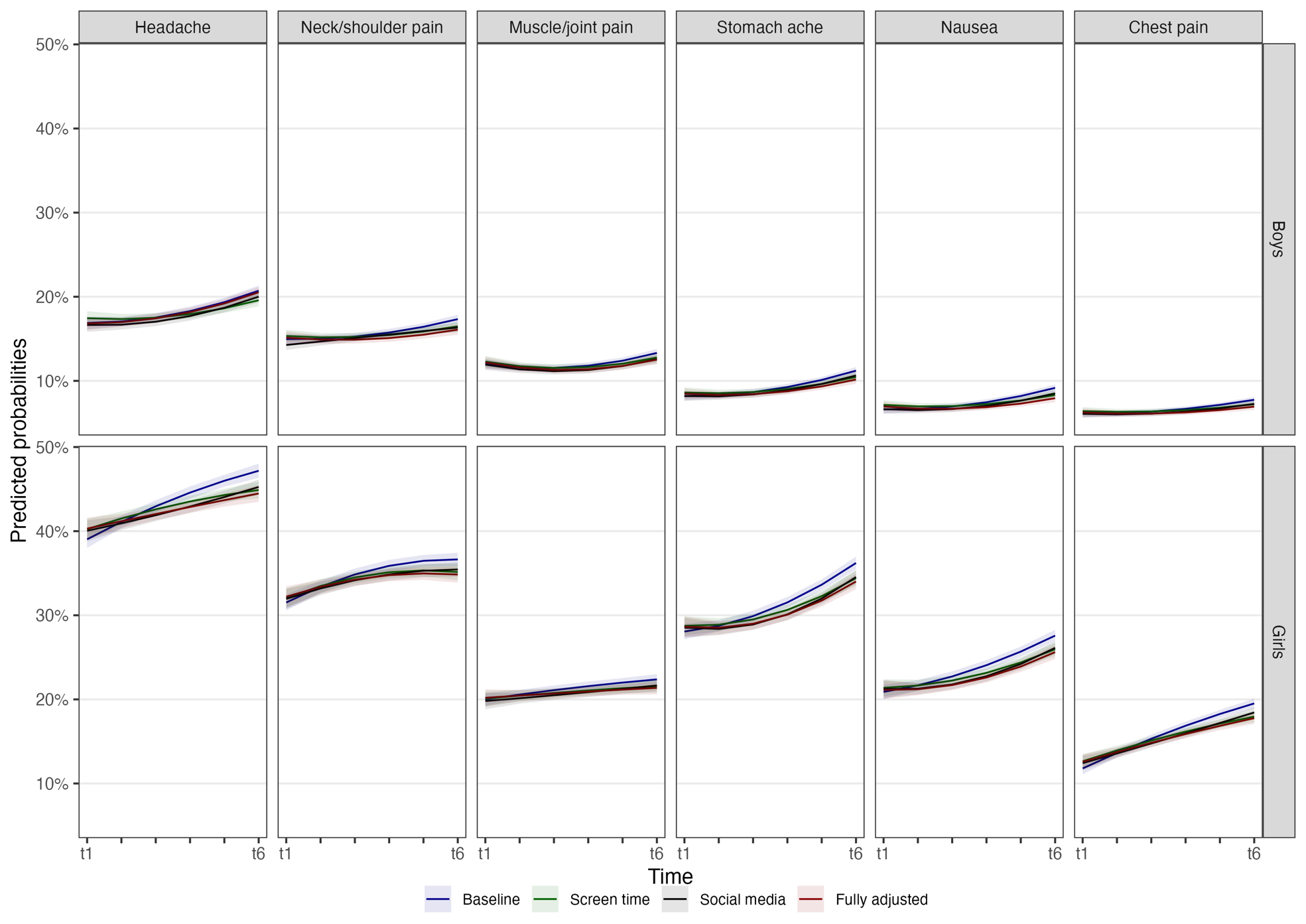


Note. Predicted growth curves from multilevel linear probability models with a quadratic slope for each health complaint. t1 = 2014, t6 = 2019. Baseline model is adjusted for age (ref = 16). Screen time = Baseline model + screen time (between and within effects). Social media = Baseline model + social media use (between and within effects). Fully adjusted = Baseline model + screen time + social media + physical activity (at the between and within level).**.**
